# The 2017 human monkeypox outbreak in Nigeria—Report of outbreak experience and response in the Niger Delta University Teaching Hospital, Bayelsa State, Nigeria

**DOI:** 10.1371/journal.pone.0214229

**Published:** 2019-04-17

**Authors:** Dimie Ogoina, James Hendris Izibewule, Adesola Ogunleye, Ebi Ederiane, Uchenna Anebonam, Aworabhi Neni, Abisoye Oyeyemi, Ebimitula Nicholas Etebu, Chikwe Ihekweazu

**Affiliations:** 1 Department of Internal Medicine, Infectious Disease Unit, Niger Delta University Teaching Hospital, Okolobiri, Bayelsa State, Nigeria; 2 Nigerian Centre for Disease Control, Abuja, Nigeria; 3 Department of Paediatrics and Child Health, Niger Delta University Teaching Hospital, Okolobiri, Bayelsa State, Nigeria; 4 Bayelsa State Ministry of Health, Bayelsa, Nigeria; 5 Department of Community Medicine, Niger Delta University Teaching Hospital, Okolobiri, Bayelsa State, Nigeria; Federal Teaching Hospital Abakaliki, Ebonyi State, NIGERIA

## Abstract

**Background:**

In September 2017, Nigeria experienced a large outbreak of human monkeypox (HMPX). In this study, we report the outbreak experience and response in the Niger Delta University Teaching Hospital (NDUTH), Bayelsa state, where the index case and majority of suspected cases were reported.

**Methods:**

In a cross-sectional study between September 25^th^ and 31^st^ December 2017, we reviewed the clinical and laboratory characteristics of all suspected and confirmed cases of HMPX seen at the NDUTH and appraised the plans, activities and challenges of the hospital in response to the outbreak based on documented observations of the hospital’s infection control committee (IPC). Monkeypox cases were defined using the interim national guidelines as provided by the Nigerian Centre for Disease Control (NCDC).

**Results:**

Of 38 suspected cases of HMPX, 18(47.4%) were laboratory confirmed, 3(7.9%) were probable, while 17 (18.4%) did not fit the case definition for HMPX. Majority of the confirmed/probable cases were adults (80.9%) and males (80.9%). There was concomitant chicken pox, syphilis and HIV-1 infections in two confirmed cases and a case of nosocomial infection in one healthcare worker (HCW). The hospital established a make-shift isolation ward for case management, constituted a HMPX response team and provided IPC resources. At the outset, some HCWs were reluctant to participate in the outbreak and others avoided suspected patients. Some patients and their family members experienced stigma and discrimination and there were cases of refusal of isolation. Repeated trainings and collaborative efforts by all stakeholders addressed some of these challenges and eventually led to successful containment of the outbreak.

**Conclusion:**

While the 2017 outbreak of human monkeypox in Nigeria was contained, our report reveals gaps in outbreak response that could serve as lessons to other hospitals to strengthen epidemic preparedness and response activities in the hospital setting.

## Introduction

Monkeypox is a rare zoonotic viral infection caused by the monkeypox virus, a genus of Orthopoxvirus in the Pox family of viruses that includes smallpox, cowpox and vaccinia viruses.[1] The disease was first reported in 1958 among laboratory monkeys but the first human case was reported in 1970 in a 9 month old boy living in the Democratic Republic of Congo (DRC).[2] Human monkeypox is a smallpox like illnesses characterized by a prodrome of fever and malaise accompanied by progressive appearance of vesiculopustular skin lesions. The morbidity and mortality of human monkeypox is however much milder than that of smallpox.[3]

The West African and Congo Basin Clades are the two clades of monkeypox virus known to cause endemic disease in the DRC and sporadic outbreaks in many parts of Central and West Africa (including Sierra Leone, Nigeria, Cote d’voire), as well as in the United States of America.[4] The Congo Basin clade of virus is responsible for more severe outbreaks that are reported in most parts of Central Africa while the West Africa Clade accounts for milder outbreaks from other parts of the world.

Human monkeypox is believed to have multiple animal reservoirs including squirrels, Gambian rats and other primates.[5] The virus is primarily transmitted by direct contact with infectious secretions from animals via handling of infected animals or consumption of poorly cooked bush meat.[1,5] Person-to-person secondary transmission may occur mainly via respiratory droplets, direct contact with infected secretions of patients or from contaminated patient environment. Prior outbreaks suggest greater potential for person-to-person transmission in Congo Basin than West African Clade. [5,6]

Between 1970 and 2017, Nigeria reported a total of 3 cases of human monkeypox; one case in 1970 and two cases in 1978.[2] In September 2017, there was a re-emergence of the largest outbreak of the West African Clade of human monkeypox in Nigeria with 228 suspected cases, (60 confirmed cases) reported in 24 out of the 36 states of the country.[7] The first case and majority of suspected and confirmed cases were reported in Bayelsa state and managed at the Niger Delta University Teaching Hospital, Bayelsa state. In this paper, we report the outbreak experience and response by this tertiary hospital.

## Methods

### Study design

The cross-sectional study was undertaken between 20^th^ September and 31^st^ December 2017 using qualitative and qualitative data collection methods. We reviewed the clinical characteristics of all suspected and confirmed cases of monkeypox seen at the hospital during the study period, including cases of false alarms. In addition, we reviewed and appraised the plans, activities and challenges of the hospital in response to the outbreak throughout the study period.

### Study site and setting

The study was undertaken in the Niger Delta University Teaching Hospital (NDUTH), a 200-bed hospital situated in Bayelsa state, in the Niger Delta Region of Nigeria. The hospital provides both comprehensive general medical and surgical care as well as specialist care in the major fields of healthcare.

Bayelsa state was the first state to report the 2017 human monkeypox outbreak in Nigeria as the index patient was first diagnosed and managed at the NDUTH. At the onset of the outbreak, the hospital had no designated isolation facility for management of suspected infectious disease outbreaks. However, there was an existing infection control committee consisting of an infectious disease specialist, a public health physician, microbiologists and an infection control nurse, among others.

### Definition of terms

At the onset of the outbreak, the case definition of human monkeypox was defined broadly to ensure no potential case was missed. Consequently, any patient with vesiculopustular lesions with or without fever was initially considered a suspected case of monkeypox case. About two weeks after the first case was reported, the NCDC provided standardized case definition including:

Suspected case of monkeypox: Any person presenting with a history of sudden onset of fever, followed by a vesiculopustular rash occurring mostly on the face, palms and soles of feet.

Confirmed Case: Any suspected case with laboratory confirmation (Positive IgM Antibody and PCR or Virus isolation). Positive PCR alone was suggestive of a confirmed case independent of IgM results.

Probable: Any suspected case with epidemiological linkage with a confirmed case in whom laboratory testing could not be carried out.

For this report, we defined false alarm as: any patient with skin rash typical of other diseases, but wrongly referred to our hospital as a suspected case of monkeypox.

### Laboratory testing

Blood and skin samples were collected from patients following strict infection control guidelines and in accordance with the NCDC interim guidelines on laboratory diagnosis of human monkeypox. At least two specimens (blood, swab or crust) were collected from each patient for laboratory investigation by real time polymerase chain reaction (PCR), serology and culture. The genomic characteristics of human monkeypox from the 2017 Nigeria outbreak have been published.[8] Suspected cases seen at our centre were confirmed as human monkeypox by IgM serology and PCR. All laboratory investigations for monkyepox virus infection were done by the Nigerian Centre for Disease Control in Collaboration with its international partners. Details of laboratory testing procedure have been previously described. [7,8]

As possible and depending on the differential diagnosis, suspected cases also had skin biopsy, chicken pox serology, hepatitis B and C serology, syphilis serology (VDRL), HIV testing and complete blood count.

### Data collection

Using a standardized data entry form, we collected demographic, clinical and laboratory data of all study participants.

Beginning September 2017, the Infection Prevention and Control (IPAC) committee documented the hospitals plans, activities and challenges during and after the outbreak. At the end of the outbreak, we appraised the hospital’s response using the Interim National Guidelines for Monkeypox Outbreak standard by the NCDC.[9] Specifically, we retrospectively appraised coordination of the response, establishment of monkeypox-response committee, education and training of HCWs, provision of IPC resources and isolation precautions, as well as screening and management of suspected monkeypox patients.

We also documented the opinions of healthcare workers (HCW) during the outbreak through focus group discussions undertaken during monkeypox-education and training programmes. All members of the monkeypox response team also documented the views and behaviour of suspected cases, their family members and HCW during individual discussions and during evaluation of cases referred to the monkeypox-response team. All documented opinions and behaviour were reviewed and domains relating to: perceptions about monkeypox disease, willingness to participate in the monkeypox response; screening and case management of suspected cases; response to establishment of monkeypox-isolation ward; and post-outbreak behavior, were identified.

Ethical approval for the study was obtained from the NDUTH institutional ethical review committee. Verbal consents were obtained from all study participants or their guardians as applicable, including consents for clinical photographs.

## Results

### The index patient

**Fig 1:**
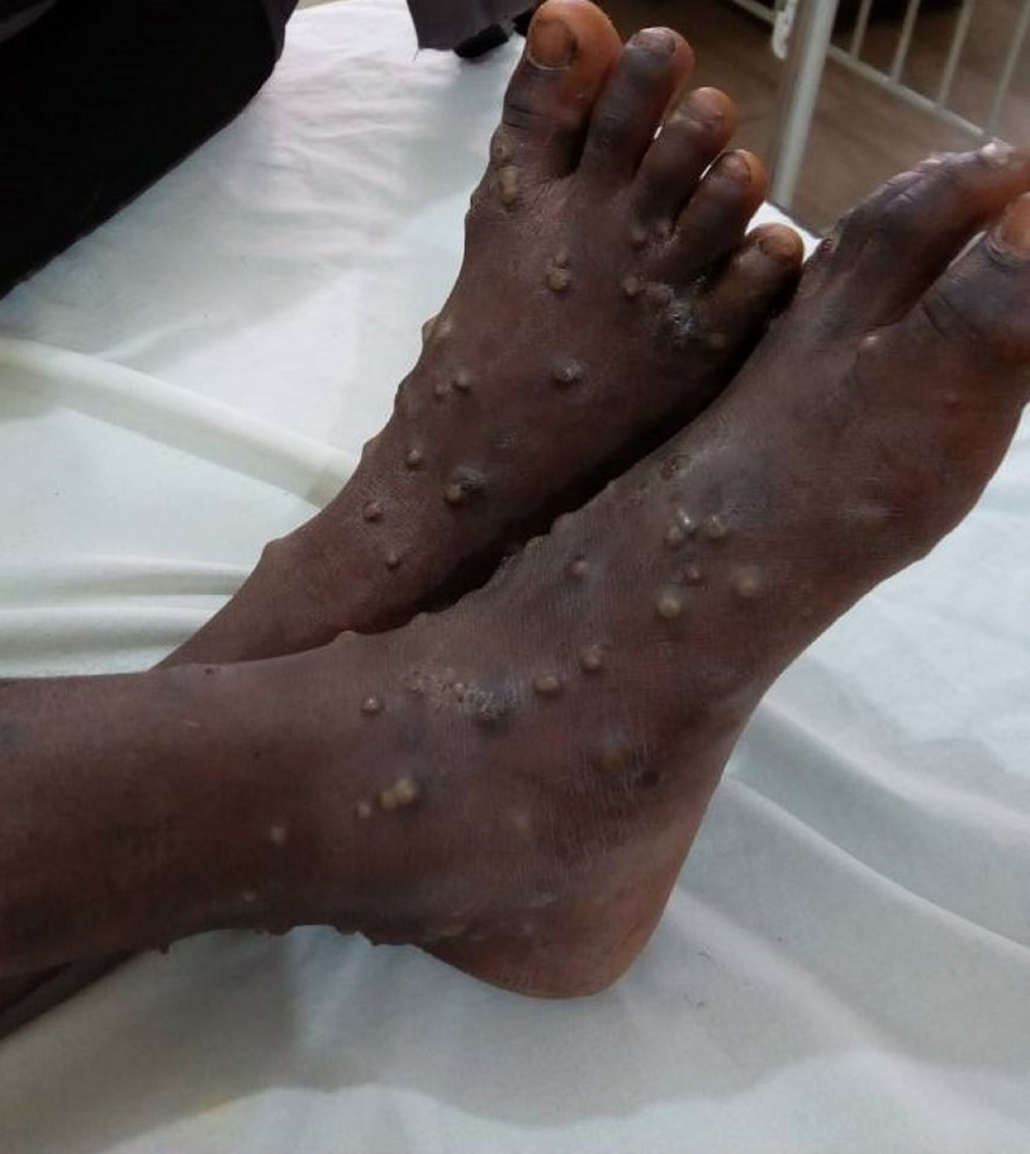
Vesiculopustular lesions on feet of index monkeypox case.

On Friday 22nd September 2017, an 11year old boy was referred to NDUTH from a private health facility with an 11-day history of fever, malaise and progressive appearance of vesiculopustular rashes on his skin (Fig 1), oral and nasal mucosa, with associated generalized lymphadenopathy. The admitting clinicians initially considered chicken pox but in view of the nature of the skin lesions and the persistence of symptoms, human monkeypox was later considered as a possibility. Two days after admission, hospital, state and national authorities were notified for further investigation and confirmation of this suspected case of human monkeypox.

It was later observed that four other family members (the index patients’ brother, uncle, father and mother) had also developed similar skin lesions (two before and two after the onset of symptoms in the index case), but unlike the index case the symptoms were mild and resolved spontaneously without requiring hospital admission.

### Clinical and laboratory characteristics of suspected and confirmed cases

A total of 38 suspected cases of human monkeypox (representing 16.7% of 228 cases across the country) were seen at our hospital, including 29 adults and 9 paediatric cases. Of the 38 suspected cases, 21 (representing 35% of 60confirmed/probable cases across the country) met the case definition for human monkeypox, including 18 laboratory-confirmed and three probable cases. Two of the 21 cases had laboratory evidence of concomitant chicken pox (by PCR/serology). Seventeen cases did not fit the case definition for confirmed or probable cases of monkeypox. Of these 17 cases, 13 (76.5%) had clinical features suggestive of chicken pox (three were laboratory confirmed), three were syphilis VDRL positive and one HIV-infected patient was confirmed as a case of cutaneous lymphocytosis by histology of skin biopsy lesions.

**Fig 2:**
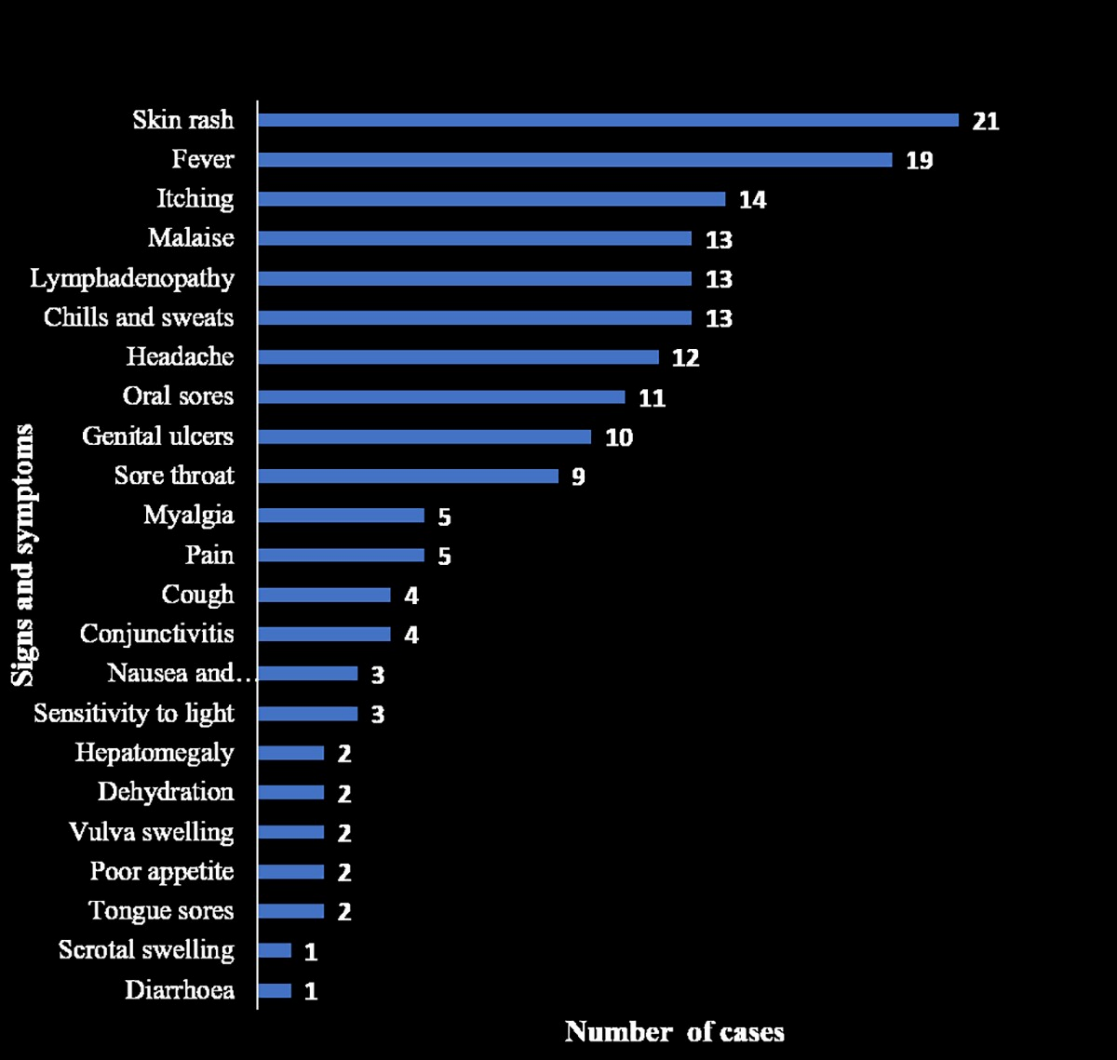
Clinical signs and symptoms of 21 cases of human monkeypox seen at NDUTH. The most common clinical features were fever, skin itching, headache and lymphadenopathy.

The frequency of clinical characteristics of the 21 cases of human monkeypox are shown in Fig 2. The 21 cases were aged 6 to 45years (median 29 years, interquartile range of 22 and 33years); 17(80.9%) were adults, and 17(80.9%) were males. Of the 21 cases, 8 (38.1%) were students, 10 were traders/artisans (47.6%), 1(4.8%) each was a farmer and HCW. The HCW was actively involved in the management of suspected and confirmed cases.

The major clinical symptoms of the 21 cases other than rash were; fever (90.5% of cases), skin itching (66.7%), headache (61.9%) and lymphadenopathy (61.9%). Thirteen (61.9%) of the 21 cases were hospitalized, while 8 (38.1%) cases were managed on out-patient basis. One case was in the first trimester of pregnancy at presentation but later had PROM and spontaneous vaginal delivery of a macerated fetus.

Additional laboratory investigations were possible in only eight (38.1%) of the 21 cases. Two of these eight patients were HIV-1 positive (CD4 cell count of 354 and 280 cells/ul) and another two patients were VDRL reactive. Both HIV positive cases had over 100 skin lesions associated with genital ulcers, which resulted from ulceration of monkeypox lesions affecting the genitalia. One case had thromobocytopaenia (platelets-78 x 10^3^/l). Haemoglobin levels (range- 10.7–16.2g/dl), White blood cell count (4.3–11.6 x 106/l) and differentials (neutrophils- 41–60%, lymphocyte- 33–53%, Basophils/Eosinophils- 2–23%) were all within normal limits. HbsAg and HCV antibodies were negative in all 8 patients. Other than a case of suicide in one case, there were no monkeypox related mortalities.

### Plans and activities of the hospital in response to the monkeypox outbreak

About a week after recognition of the outbreak, a team from the NCDC arrived in Bayelsa state to partner with the state government in the outbreak response and control. The NDUTH was then designated as the treatment centre for all suspected cases of monkeypox. The hospitals 12-bed medical ward was converted to the designated isolation facility for all adult cases requiring admission. This ward was to accommodate male and female patients in different sections with separate toilet facilities.

The state government made a pronouncement to treat all suspected cases free and to cater for their welfare and feeding if admitted in the hospital.

An NDUTH monkeypox response committee was constituted, including clinical teams for case management, waste management and laboratory investigations. The monkeypox response team was mandated to coordinate the hospital preparations and response to the outbreak with support and partnership from the teams from the NCDC and the Bayelsa state ministry of health.

Hospital-wide sensitization workshops were organized on various days to inform and educate staff about monkeypox. Thereafter, clinicians were trained on various aspects of standard precautions of infection control, use of personal protective equipment and healthcare waste management. There were practical demonstrations on hand hygiene and use of personal protective equipment (PPE). There were separate training sessions for health attendants and cleaners, laboratory workers and doctors/nurses. The sensitization and training activities provided a platform to obtain views of HCWs about the outbreak and to provide clarifications as necessary.

A monkeypox clinical data entry checklist was developed to ensure uniform documentation of all relevant clinical signs, symptoms and laboratory findings of all suspected cases. All HCWs were asked to be vigilant and to immediately notify the hospital’s response team of any suspected case. An HCW-case management contact list was provided to document names of all HCWs directly involved in case management, date and time of contact, as well as type of contact. All HCWs involved in case management were thereafter advised to report to the response team if they developed fever or rash.

### Opinions and behaviors of patients and relatives during outbreak

Patients and their relatives expressed diverse views and behavior during the outbreak. Majority expressed fear and anxiety over facing stigma and discrimination from hospital staff, members of the community and family members. Before intervention by the case management team, some of the patients referred to the hospital with skin rash were either avoided or abandoned by some HCWs. A few patients(n = 4) were worried over potential disfigurement, as they were uncertain if disease was curable and if skin lesions would resolve completely. Three patients and their family members initially refused isolation, believing that they could face greater stigma and discrimination if members of their community knew that they were admitted in the isolation ward. This fear of isolation ward was allayed after counselling by the state and hospital’s response teams. Two cases who had other confirmed cases among family members felt guilty that they were responsible for transmission of the disease to their family members.

### Opinions and behaviors of healthcare workers during outbreak

Following the identification of the index case as a suspected case of monkeypox, many HCWs expressed fear of contracting the infection. Many were afraid that they would face stigma and discrimination if they were part of the case management team. Some outrightly refused to participate in case management and others requested for incentives to be part of the team. The fear among HCWs was particularly heightened when a doctor managing the index patient developed a rash and had to be isolated for monkeypox evaluation. Following repeated training sessions many HCWs subsequently volunteered to be part of the response. However, a few volunteered to be part of the team only after they were promised monetary incentives.

### False alarms

During the outbreak, the hospital reported 21 cases of false alarms. These include: typical cases of chicken pox (n = 8); mosquito bites (n = 2); molluscum contagiosum (n = 2); impetigo (n = 1); facial acne (n = 3); tinea corporis (n = 2); psoriasis(n = 1); scabies (n = 1) and petechiae rash in a patient with bleeding disorder (n = 1).

### Media and public attention during the outbreak

As soon as the outbreak of suspected human monkey pox was officially declared on 5^th^ October 2017 by the Federal Ministry of Health, Nigeria, the hospital became a centre for public and media attention. There were several visits and calls to the hospital by local, national and international media organizations. There were several publications in social, print and televised media about the “strange disease” in the NDUTH. The hospital management, the NDUTH and State’s, monkeypox response teams granted several media interviews to provide information, give clarifications, allay fears and give assurances that adequate measures were in place to control and contain the outbreak.

### Challenges faced during the outbreak

**Fig 3:**
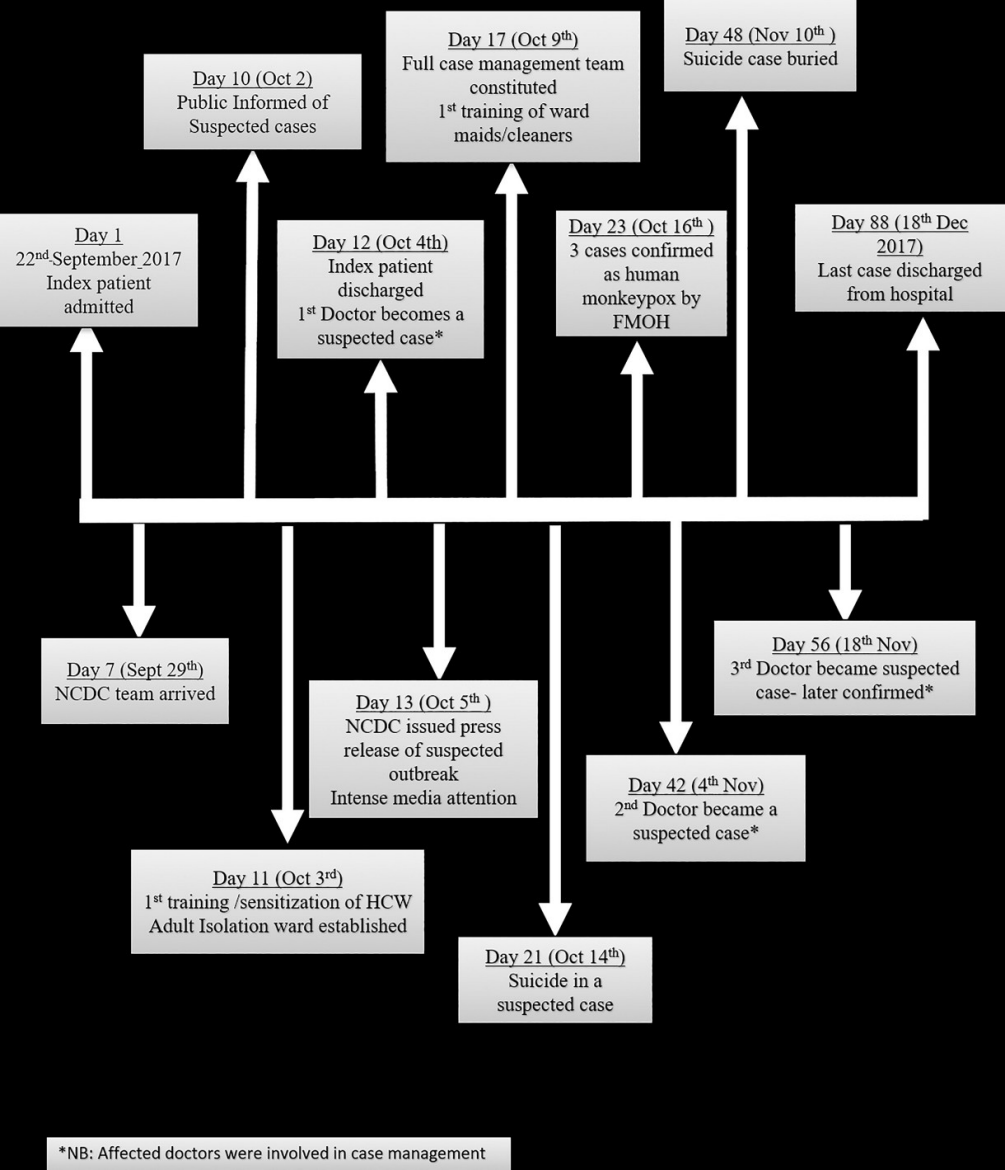
NDUTH human monkeypox outbreak response timeline. This figure illustrates the sequence of major events that occurred following the diagnosis of the index case on the first day of the outbreak and the discharge of the last case after recovery.

On appraisal of the hospital’s response, the most prominent challenge identified was the delay in confirmatory diagnosis of monkeypox due to lack of capacity of any laboratory in Nigeria to diagnose the disease at the onset of the outbreak. Other challenges identified included reluctance of HCWs to participate in the response due to fear of the disease and lack of standard isolation facilities to appropriately and safely manage cases. The death of one of the confirmed monkeypox cases by suicide was a significant challenge to the response, as it generated public misconceptions, threw up challenges related to safe burial of the corpse and elicited emotional distress among family members. This patient was later safely buried after psychological counselling and support were provided to the family members of the deceased. Fig 3 summarizes the chronology of major events that occurred after the index case was admitted to the hospital to the time the last case was discharged.

## Discussion

The admission of the first suspected case of human monkeypox in our hospital in September 2017 and the subsequent laboratory confirmation of this case signaled the reemergence of West African Clade of human monkeypox in Nigeria, after 38years since it was last reported. The overall epidemiology and molecular characteristics of the 2017 human monkeypox outbreak in Nigeria have been previously reported.[7,8] Remarkably, as observed among cases in this report, the Nigeria outbreak was characterized by predominant infection among young adult males and significant person-to-person secondary transmission. [7,8] These findings differ from previous reports of the West African Clade of human monkeypox where children below 10 years of age comprised 83% of the cases[2] and secondary transmission was observed to be rare. [2,10]

The clinical manifestations of human monkeypox have been shown to be influenced by the route of infection with animal source and complex invasive infections associated with more severe symptoms than person-to-person transmissions due to skin contact. [11,12] While the clinical findings in our study are like other studies [2,13,14], it is noteworthy that a substantial number of our cases who were young adults in their reproductive age presenting with genital ulcers, as well as concomitant syphilis and HIV infection. Although the role of sexual transmission of human monkeypox is not established, sexual transmission is plausible in some of these patients through close skin to skin contact during sexual intercourse or by transmission via genital secretions. The role of genital secretions in transmission of human monkeypox, however deserves further studies. Our findings also suggest that HIV-infection might negatively influence the morbidity of human monkeypox as patients with HIV had more severe skin lesions associated with genital ulcers as compared with HIV-negative individuals. Nigeria has a HIV prevalence of 3.4% while the prevalence in Bayelsa state is 2.7%.[15] In view of relative endemicity of HIV in Nigeria, the emergence of HIV-monkeypox coinfection and associated co-morbidities might pose significant clinical and public health challenges in the preventing and control of both diseases in Nigeria.

The hospital’s experience and response to this outbreak reveal various areas of strengths and weaknesses that could serve as lessons for similar hospitals facing epidemics. Although monkeypox had not been seen or managed in any hospital in Nigeria for 38years, the lack of monkeypox diagnostic capacity in the country and the absence of a suitable isolation facility for management of suspected cases at our hospital were significant challenges that caused uncertainties, delays in case management, anxiety and fear among patients, family members and healthcare workers alike. The emergence and role of fear and anxiety in infectious diseases outbreaks have been previously reported.[16] In the 2014 Ebola outbreak in Nigeria for instance, fear among HCWs and the general population played a role in shaping the nature of response to the outbreak.[17] Similarly, we observed various behavioural and emotional responses to the monkeypox outbreak among our staff, patients and their family members, which resulted in avoidance of suspected patients, refusal to accept isolation and suicide in one case. The confirmation of infection in one of the HCW involved in case management supports previous reports of nosocomial transmission of monkeypox.[18] Although, the route of infection in the HCW could not be ascertained, a breach in infection control measures is possible. The occurrence of healthcare-associated monkeypox infections emphasizes the need for strict observance of infection control measures during care of suspected monkeypox cases.

The eventual successful containment of the monkeypox outbreak in our hospital and in Nigeria in general could be attributed to strong commitment and partnership by institutional and political leadership at the hospital, state and federal government levels. The presence of an existing IPAC committee in the hospital led by an infectious disease physician facilitated prompt expert case management and strengthened infection control activities during the outbreak. The provision of free treatment and meals to all suspected patients by the state government enabled early isolation and effective treatment of suspected cases who otherwise would have stayed at home putting others at risk. The coordinating and supervisory role of the federal government lead by the Nigerian Centre for Disease Control ensured laboratory confirmation of diagnosis, support of the hospital in training of healthcare workers, provision of infection control resources and case management. Overall, it was evident that all stakeholders displayed ownership of the outbreak response and provided the required leadership and cooperation that led to the successful containment of the outbreak.

Several lessons were learnt from our experience. Our report shows that exemplary clinical and political leadership, capacity building of HCWs, provision of adequate IPAC resources and incentives, were good motivators that positively influenced prompt and sustainable response to the outbreak by all stakeholders. This outbreak also revealed that other than an IPAC team, hospitals ought to have an existing multi-disciplinary team to adequately respond to any outbreak. Such a team should include a broad-based clinical team for case management (including doctors of various fields, pharmacists and nurses), a laboratory team (including pathologists and laboratory scientists), a safe burial team (including pathologists and morticians) as well as a counselling and social support team (including a psychiatrist, social workers and health counsellors) and support services (including cleaners and health attendants).

Outbreak communication is an essential component of every successful outbreak response.[19] The unprecedented public and media attention on the hospital during the outbreak revealed several lessons. Firstly, as part of outbreak response strategy and to avoid misinformation and misconceptions, hospitals ought to enlighten all its staff against sharing patient information in the social media or unauthorized communication with the media. Secondly, only one or two designated and trained staff should be authorized to communicate with the media on issues related to the outbreak to avoid contradictory and unverified reports. Ultimately, it is necessary for hospitals with potential to face epidemics to be routinely trained on media management and outbreak communication.

There are some limitations in this study. First, we largely used documentary evidence from the IPAC members as basis for making some conclusions in this study. While recall and inter-observer biases are possible, we still believe our findings were valid as they are plausible and comparable to findings reported in other outbreaks. Second, our results were based on a single hospital experience and may not necessarily be generalizable to other hospitals in Nigeria.

In conclusion, this study has shown that hospitals most often represent the first location where outbreaks are identified. While concerted and collaborative efforts by all stakeholders led to the containment of the monkeypox outbreak in Nigeria, our report reveal gaps in outbreak response that could serve as lessons to other hospitals in Africa and around the world to strengthen epidemic preparedness and response activities in the hospital setting.

## Supporting information

S1 Table Clinical features of 21 human monkeypox cases seen at NDUTH. (XLSX)
